# ADAPT as a triage system in a Danish Emergency Department

**DOI:** 10.1186/1757-7241-20-S2-P3

**Published:** 2012-04-16

**Authors:** Martin Bøhme Rasmussen, Christian Backer Mogensen

**Affiliations:** 1 Emergency Department, Sygehus Lillebælt, Kolding, Denmark

## Background

ADAPT was introduced in the Emergency Department (ED) Kolding Hospital, June 2010. It was the first step of implementing a standardized and systematic way of making a risk assessment of every patient admitted. By being simple, reproducible and easy useable in the everyday of an ED it should secure that the patients were seen and treated in the correct order.

In this study we wished to illustrate how the triage categories were distributed among the specialties, to analyse whether there was a connection between triage category and duty shifts and weekday for admission and connection between triage category and main complaints.

## Methods

The project consisted of two parts with descriptive retrospective studies and non-blinded journal reading and collecting of data. Patients admitted in the specialties internal medicine, abdominal surgery, vascular surgery and orthopaedic surgery were included in the study.

In the period 01.06.10-31.12.10 3,876 patients were admitted and triaged by ADAPT. We divided the triage categories into two groups: serious (red/orange) and non-serious (yellow/green/blue).

A sample of 500 random picked journals was examined and triage category and chief complaint were registered.

## Results

The patients were triaged red, orange, yellow, green and blue in 0.8%, 16.4%, 41.1%, 41.2% and 0.6% of the cases respectively.

Among 3,339 patients triaged with information about time of the day 14.5% had a serious triage during dayshift (8 am- 4 pm), 21.5% during evening shift (4 pm-midnight) and 20.5% during nightshift (midnight-8 am), lower percentage during day shift (p=0.0002, Chi square test).

Among 3,876 patients triaged with information about day of the week 16.3% had a serious triage during working days and 19.8% during weekends (p=0.005, Chi square test).

Gastrointestinal complaint (39.4%) was the most common followed by infection/fever (12.4%), extremity swelling/pain (8.8%) and chest pain (6%).

## Conclusion

A minority of the patients needed immediate treatment but there was a significant higher occurrence of serious triaged patients during evening and night and in weekends. Gastrointestinal complaint was the most frequent used main complaint.

